# Nutritional Status and Caregiver Knowledge in Pediatric Cystic Fibrosis Management

**DOI:** 10.3390/nu18183034

**Published:** 2026-09-16

**Authors:** Hadil S. Subih, Nour Khamis, Belal S. Obeidat, Nisreen Rumman, Linda Alyahya

**Affiliations:** 1 Department of Nutrition and Food Technology, Faculty of Agriculture, Jordan University of Science and Technology, P.O. Box 3030, Irbid 22110, Jordan; 2 Department of Animal Production, Faculty of Agriculture, Jordan University of Science and Technology, P.O. Box 3030, Irbid 22110, Jordan; 3 Pulmonary Clinical Medical Department, Caritas Baby Hospital (CBH), Bethlehem, Palestine; 4 School of Pharmaceutical Sciences, Universiti Sains Malaysia, Gelugor 11800, Malaysia

**Keywords:** cystic fibrosis, pediatric nutrition, caregiver nutritional knowledge, dietary adherence, nutritional status, vitamin D, FEV1, Palestine

## Abstract

Introduction: Cystic fibrosis (CF) is a rare, life-limiting genetic disorder characterized by abnormally thick mucus secretions that obstruct the respiratory, gastrointestinal, and reproductive tracts. Globally, CF affects an estimated 70,000 to 100,000 individuals. This study investigated nutritional awareness and dietary management among caregivers of pediatric CF patients. Specifically, it evaluated parental nutritional knowledge, caregiving behaviors, and patients’ actual nutrient intake and diet quality relative to established clinical guidelines. Methods: A cross-sectional study was conducted with a representative sample of 50 parents and their children with CF (aged 4–18 years) receiving specialized care at Caritas Baby Hospital in Bethlehem, Palestine. Data collection utilized a comprehensive parental questionnaire alongside a 3-day food diary completed by participants. Objective clinical status and biochemical markers were extracted directly from patients’ medical records. CF-specific recommendations were used where available, and general pediatric reference values were used otherwise. Results: A significant disparity (*p* ≤ 0.05) was observed between patients’ actual dietary intake and the recommended daily allowances for protein, carbohydrates, zinc, and calcium; however, intakes of sodium and iron did not differ significantly from guidelines. Higher patient adherence to dietary recommendations was significantly associated with superior lung function (measured via FEV1) and optimal serum albumin levels (*p* ≤ 0.05). Notably, 38% of the study sample were classified as underweight, and 76.9% of children with available 25(OH)D measurements (30/39) exhibited concentrations below 30 ng/mL. Clinical and biochemical measurements were not available for all participants. Caregiver nutritional knowledge was not significantly associated with the measured clinical or biochemical outcomes, whereas better patient dietary adherence was associated with higher FEV1% predicted and serum albumin concentrations. Conclusion: The high prevalence of underweight status and micronutrient deficiencies highlights a critical need for targeted nutritional education and clinical counseling programs. Enhancing awareness among patients and parents is essential to correct dietary deficits, optimize growth, and improve long-term health outcomes.

## 1. Introduction

Cystic fibrosis (CF) is a life-threatening autosomal recessive disease affecting many body systems. The disease is caused by mutations in the gene for the CF transmembrane conductance regulator (CFTR), which is a type of protein that controls the transport of chloride ions through epithelial cell membranes. This defect results in dysfunction of the CFTR protein with abnormal ion concentrations, leading to thick mucus secretions in the digestive, respiratory, and reproductive tracts [1].

The disease was discovered in the 1930s [2]; about 70,000 to 100,000 people worldwide are suffering from it [3]. The strongest recent clinical study is Salah et al. [4] (2023), who conducted a study at Caritas Baby Hospital (CBH), Bethlehem. The study included 77 CF patients from 58 families who attended the pediatric pulmonology clinic between January and May 2017. The patients were from the West Bank. Caritas Baby Hospital is recognized in the West Bank as the first destination for CF pediatric patients.

CF is a multisystem genetic disorder caused by pathogenic variants in the CFTR gene [5]. Although CF is relatively uncommon worldwide, it imposes a substantial clinical burden and requires lifelong multidisciplinary care. Published epidemiological data on CF in Palestine remain limited; therefore, local estimates should be interpreted cautiously unless confirmed by a population-based registry or other systematic surveillance source.

Diagnosis of CF recommends evaluating CFTR function with a sweat chloride test; patients who screen positive can be diagnosed with CF after a test showing a high level of sweat chloride concentration (>60 mmol/L) [6]. Diagnosis of the disease is referred to as a ‘family diagnosis’ because controlling it involves day-to-day treatments that require patients and their families to manage multiple regimens [7]. Extensive daily treatment regimens and follow-up have extended the CF median predicted survival from a few months after diagnosis to 38 years of age, according to the United States Cystic Fibrosis (US CF) Foundation (2008 to 2012) [2]. One of the major reasons that contributed to this increase in survival is the understanding of the role of nutrition in CF patients’ health [8]. Recent registry data from the Cystic Fibrosis Foundation Patient Registry indicate substantial improvements in survival in CF; for individuals born between 2021 and 2025, the predicted median age of survival is approximately 66 years [9]. CF is diagnosed using clinical and/or screening findings followed by evidence of CFTR dysfunction using contemporary diagnostic criteria [10]. Because nutritional impairment is a major component of CF morbidity, nutritional assessment and individualized dietary management are integral to comprehensive care [11].

About 85% of CF patients have pancreatic insufficiency linked with nutrient malabsorption and frequently undernutrition [12]; there is a strong link between good nutritional status and better lung function, leading to longer survival and better clinical results [13,14]. Children with CF may require increased energy and protein intake to compensate for malabsorption, chronic inflammation, increased work of breathing, and increased metabolic demands [15]. Pancreatic insufficiency and fat malabsorption also increase the risk of deficiencies of fat-soluble vitamins, particularly vitamins A, D, E, and K, as well as selected minerals. Therefore, dietary assessment should consider both energy and macronutrient adequacy and the risk of micronutrient insufficiency [15]. Thus, nutritional education with the treatment can be one of the ideal goals for patients’ health [16]. Treatment for such a chronic disease is often time-consuming, and adherence to such treatment is a major challenge [12]. Growth indicators, including BMI-for-age, weight-for-age, height-for-age, and longitudinal growth trajectories, are important components of nutritional monitoring in pediatric CF and are related to overall clinical status [15]. Caregivers play a central role in meal planning, food selection, pancreatic enzyme administration, vitamin supplementation, and reinforcement of high-energy dietary recommendations. Thus, caregiver nutritional knowledge may influence the extent to which recommended dietary practices are implemented at home [17], especially with poor appetite, gastrointestinal symptoms, food preferences, socioeconomic constraints, limited access to specialized dietetic services, and caregiver burden. Parents at home are involved in managing the treatment, providing the health services needed (e.g., intravenous therapy, medications, and physiotherapy) [18], and giving suitable supervision for their children that can increase their adherence to nutritional recommendations by influencing their children’s food choices [19].

The objectives were to assess caregiver CF-specific nutritional knowledge, evaluate pediatric adherence to prescribed nutritional recommendations, characterize dietary nutrient intake, and examine associations of adherence and caregiver knowledge with anthropometric, biochemical, and pulmonary measures. We hypothesized that higher caregiver knowledge and higher patient dietary adherence would be associated with better nutritional and clinical indicators, including BMI-for-age, nutrient adequacy, serum 25(OH)D/albumin, and FEV1.

## 2. Materials and Methods

### 2.1. Study Design and Participants

A cross-sectional study was conducted between January and November 2022. It included 50 parents of CF patients and 50 of their CF children aged 4–18 years who were receiving follow-up care throughout the clinics at Caritas Baby Hospital (CBH) in Bethlehem city, Palestine. Parents who participated in our study were interviewed by the researcher for the purpose of the study and filled out a questionnaire of 43 questions about their CF children and how they manage their disease. The study protocol was approved by the Institutional Review Board of Jordan University of Science and Technology (IRB # 13/110/2017; approved on 16 November 2017) and the Scientific Research Committee of Caritas Baby Hospital. Informed signed consent was obtained from each participant who was willing and eligible to participate in the study.

### 2.2. Questionnaire Design and Validity

Data collection was administered through structured, face-to-face interviews by a trained nutritionist with at least one parent or primary caregiver. The specialized questionnaire was developed to evaluate parental caregiving and nutritional practices and consisted of four core sections.

Through this instrument, investigators gathered detailed data across multiple domains:

- Sociodemographic Profile: 7 questions included caregiver age, gender, number of siblings with CF, formal educational attainment, marital status, and parental consanguinity (whether the parents are biologically related).
- Pediatric Lifestyle and Physical Activity: 11 questions were asked to assess routine daily behaviors and physical exertion levels of the patient.
- Dietary Habits: Household dietary patterns, specifically the frequency of main meals and snacks consumed by the child daily. The patients were given a 3-day food record sheet (two weekdays and one weekend) to write down everything they eat or drink.
- Parents’ nutritional knowledge: 12 questions were asked, and respondents were asked to answer each question carefully. If respondents answered all 12 questions correctly, they would receive a 100% score. This included questions such as enzymes taken before or after food and drink.
- Childrens’ adherence to good nutritional habits: 7 questions were asked to assess the level of adherence.

### 2.3. Clinical Measurements

Data on serum albumin, total protein, serum 25-hydroxyvitamin D [25(OH)D], and FEV1 were obtained from the most recent available hospital assessment. Dietary records were recorded immediately after clinical measurements were obtained. FEV1 was expressed as % predicted; clinical stability at the time of testing was not consistently documented, and FEV1 ≤ 80% predicted was used as the descriptive threshold for abnormal pulmonary function. Serum 25(OH)D was categorized as deficiency (≤20 ng/mL), insufficiency (21–29 ng/mL), and normal status (≥30 ng/mL). Clinical and biochemical measurements were not available for all participants: FEV1 data were available for 25/50 children (50%), mainly because a large share of the cohort (40% aged 4–8 years) is below the age where reliable spirometry is feasible.25(OH)D for 39/50 (78%), total protein for 38/50 (76%), and albumin for 39/50 (78%). Percentages for these outcomes were therefore calculated using only participants with available measurements.

### 2.4. Dietary Intake

Participants were provided with a 3-day food record covering two weekdays and one weekend day. A trained nutritionist instructed parents and children to record all foods and beverages consumed in the next days at the time of consumption (Prospectively), including amounts, preparation methods, and food additives. Food models and standard measuring bowls, cups, and spoons were used to improve portion-size estimation. The completed records were entered into the Food Processor Nutrition and Fitness Analysis Software (ESHA, version 7.71, Salem, OR, USA). Parents/caregivers recorded the child’s food and beverage intake, with assistance from the child when appropriate. This approach may introduce reporting or portion-estimation error. Nutrient intakes were compared with CF-specific recommendations where available; general pediatric reference values were used only when CF-specific recommendations were unavailable. CF-specific nutritional recommendations were used where available; general-population reference values were retained only where CF-specific recommendations were unavailable and are identified accordingly. Supplements/enzymes and foods consumed outside the home were encouraged to be recorded by the parents.

### 2.5. Anthropometric Measurements

Height and weight were measured for each patient. Each patient was weighed on an electronic digital scale (kilograms), and height was measured using a stadiometer (meters). These measurements were used to calculate the body mass index (BMI) (weight (kg)/height (m^2^)). Height, weight, and BMI data were used in the growth charts (percentiles) to adjust for age and gender using the National Center for Health Statistics (NCHS) data to determine nutrition risk. According to previous publications, patients were classified as malnourished if the weight-for-age < 5th percentile, height-for-age < 5th percentile, or BMI-for-age < 10th percentile [8,20].

### 2.6. Assessment of Nutritional Knowledge and Adherence

Parental nutritional beliefs and cystic fibrosis (CF)-specific knowledge were quantified using a 12-item instrument (While comprehensive, multi-center validated tools such as the Mountain West Cystic Fibrosis Consortium Questionnaire (MWCFC-Q) and the Marciel & Quittner CF Nutrition Knowledge Questionnaire exist to map parental nutritional literacy, they often require extensive administration times or may not align perfectly with localized regional clinical protocols. Therefore, a concise, 12-item disease-specific instrument focusing heavily on critical care behaviors—such as the timing of pancreatic enzyme replacement therapy—was developed and content-revised by a panel of local experts to minimize participant burden while maintaining clinical precision within the West Bank context. The caregiver knowledge and patient adherence questionnaires were developed specifically for this study and underwent expert content review. They should therefore be considered study-specific instruments rather than fully validated psychometric tools. The score categories (<40%, 40–60%, and >60%) were prespecified for descriptive classification and do not represent validated clinical thresholds. Further studies should evaluate the reliability and construct validity of these instruments.

Respondents were required to answer every question, with items addressing critical clinical care components, such as the appropriate timing of pancreatic enzyme replacement therapy relative to food and beverage intake. Scores were calculated as percentages, where answering all 12 items correctly resulted in a perfect score of 100 percent. Similarly, pediatric adherence to recommended dietary habits was evaluated using a 7-item questionnaire, with complete adherence across all items corresponding to a score of 100 percent.

To facilitate descriptive comparison, both caregiver knowledge and patient adherence scores were stratified using the prespecified study categories:

- Poor: Scores strictly less than 40 percent.
- Good: Scores ranging from 40 percent to 60 percent.
- Very Good: Scores greater than 60 percent.

Identical percentage thresholds were applied to caregiver knowledge and patient adherence: poor < 40%, good 40–60%, and very good > 60%. These cut-offs were used as study-specific descriptive categories and should not be interpreted as validated clinical thresholds.

### 2.7. Data Analysis

Data were analyzed and processed using the Statistical Package for Social Sciences software version 19.0 (SPSS Inc., Chicago, IL, USA). Characteristics of subjects’ variables were described using frequency distribution for categorical variables and mean and standard deviation for continuous variables. A *p*-value of ≤0.05 was considered the cut-off level for statistical significance. Group comparisons were done using *t*-tests and analysis of variance (ANOVA) to examine the impact of the variables of interest. Least Significant Difference (LSD) post-hoc ANOVA was conducted to determine the differences between variable groups. Simple correlation coefficients were determined. No multivariable regression analysis was performed because of the limited sample size and incomplete availability of some clinical outcomes. The 3-day record was entered into a computerized Nutrient Analysis Program (The Food Processor Nutrition and Fitness Analysis Software (ESHA), version 7.71) for further analysis. Because multiple subgroup comparisons were performed, *p*-values close to 0.05 were interpreted cautiously. The small sizes of some age- and sex-specific subgroups also limit the robustness of parametric and post-hoc comparisons.

## 3. Results

### 3.1. Participants’ General Characteristics

Table 1 outlines the demographic and anthropometric characteristics of the 50 pediatric patients with cystic fibrosis (CF) included in the study. The cohort was evenly split between younger age groups (40% aged 4–8 years and 40% aged 9–13 years), with older adolescents (14–18 years) accounting for the remaining 20%. Females made up a slight majority of the cohort at 58%. Anthropometric evaluation revealed substantial rates of undernutrition across the sample: 38% of patients were classified as underweight based on body mass index (BMI) percentiles, while 48% fell into the underweight category based on weight percentiles alone, and 26% exhibited short stature for their age. During the study period, 50 children were eligible, 50 children were approached, and 50 child–caregiver pairs were enrolled. Dietary records were completed by all participants. Clinical and biochemical data were available for FEV1 (*N* = 25), 25(OH)D (*N* = 39), total protein (*N* = 38), and albumin (*N* = 39).

### 3.2. Patients’ Clinical and Biochemical Tests

Table 2 summarizes the available clinical and biochemical measurements. Clinical and biochemical measurements were not available for all 50 participants. FEV1 data were available for 25 children, 25(OH)D for 39, total protein for 38, and albumin for 39. Percentages for these variables were therefore calculated using the number of participants with available measurements. The tests were obtained based on the doctors’ requests, based on the symptoms and complications of the disease for every patient. Among the 25 children with available FEV1 measurements, 12 (48%) had FEV1 ≤ 80% predicted. Among the 39 children with available 25(OH)D measurements, 20 (51.3%) had 25(OH)D deficiency, 10 (25.6%) had insufficiency, and 9 (23.1%) had concentrations ≥ 30 ng/mL. Vitamin D/multivitamin supplement use and pancreatic insufficiency/PERT adequacy were not available in the present dataset. Among the 38 children with available total protein measurements, 35 (92.1%) had concentrations within the stated reference range. All children with available albumin measurements (39/39; 100%) had albumin concentrations within the stated reference range. The different denominators reflect incomplete availability of clinical and biochemical measurements. These results describe only participants with available measurements and should not be interpreted as representative of the entire *N* = 50 cohort. Genotype, pancreatic exocrine status, and CFTR-modulator use were not available in the present dataset and therefore could not be examined.

### 3.3. Demographic Profile of Caregivers

Sociodemographic characteristics of the participating caregivers (*N* = 50) showed that all caregivers were mothers. Formal education was predominantly primary (50%) or secondary (34%), with 14% having college education. Consanguinity is categorized based on the closeness of the parents’ family connection, measured through degrees of relationship. Table 3 reports 29 (58%) caregivers as having parents classified as first-degree relatives and 8 (16%) as second-degree relatives; the terminology should be interpreted according to the relationship categories used in the original questionnaire. In addition, 19 (38%) mothers reported a family history of CF. Twenty-two (44%) mothers reported caring for more than one child with CF.

### 3.4. Daily Intake of Dietary Macronutrients

Mean macronutrient contributions to total energy were 38.42 ± 7.04% for fat, 17.72 ± 4.68% for protein, and 51.17 ± 8.93% for carbohydrate. Fat did not differ significantly from the selected 40% reference (*p* = 0.12), whereas protein was lower than the 20% reference (*p* = 0.01) and carbohydrate was higher than the 40% reference (*p* = 0.01) (Table 4).

### 3.5. Daily Intakes of Calcium, Sodium, and Zinc

Table 5 presents age- and gender-stratified daily intakes of key minerals compared to standard dietary recommendations. While calcium intake in younger children (4–8 years) met guidelines, older cohorts experienced significant calcium deficits, falling well below recommended intakes in both the 9–13 age group (598.91 mg vs. 1000 mg; *p* = 0.01) and the 14–18 age group (831.49 mg vs. 1150 mg; *p* = 0.01). Daily sodium intake did not differ significantly from guidelines across any age bracket. Zinc intake significantly deviated from recommendations in specific groups, notably showing reduced levels relative to targets in older girls aged 14–18 years (7.08 mg vs. 9 mg; *p* = 0.03).

### 3.6. Daily Intake of Iron

Table 6 evaluates mean dietary iron intake across gender and age tiers relative to Dietary Reference Intakes (DRIs) for healthy pediatric populations. Iron intake did not differ significantly from the selected DRI reference values in any age/sex group (*p* > 0.05 for all comparisons). Average iron intake ranged from 9.05 mg to 15.05 mg per day in boys and 9.04 mg to 12.28 mg per day in girls.

### 3.7. Caregiver Knowledge and Patient Adherence Levels

Table 7 outlines the distribution of parental nutritional knowledge scores and patient dietary adherence levels. Caregiver knowledge averaged 44.33 ± 15.37%, with 30% classified as poor, 58% as good, and 12% as very good. Patient adherence averaged 58.57 ± 16.64%, with 10% classified as poor, 54% as good, and 36% as very good.

### 3.8. Associations Between Knowledge, Adherence, and Clinical Status

Associations between caregiver nutritional knowledge, patient dietary adherence, and clinical/biochemical parameters were examined (Table 8). Caregiver knowledge categories were not significantly associated with the measured clinical or biochemical outcomes (*p* > 0.05). Better patient dietary adherence was associated with higher FEV1% predicted (*p* = 0.05) and higher serum albumin concentrations (*p* = 0.02). Mean FEV1% predicted was 51.00 ± 19.00% in the poor-adherence group, 84.07 ± 5.72% in the good-adherence group, and 86.37 ± 6.03% in the very-good-adherence group (*p* = 0.05). These findings indicate associations only and do not establish that adherence caused better pulmonary or biochemical outcomes.

## 4. Discussion

This study examined caregiver CF-specific nutritional knowledge, patient adherence to nutritional recommendations, dietary intake, and selected nutritional and clinical indicators. Caregiver knowledge was not significantly associated with the measured clinical and biochemical outcomes. Better patient adherence was associated with higher FEV1% predicted and serum albumin concentrations; however, the cross-sectional design does not establish temporal or causal relationships.

The mean values for anthropometric indicators (stature, stature percentile, weight and weight percentile) in this study were all in the normal ranges; these normal results might be an indication of the effectiveness of the treatment and nutritional guidelines. According to previous publications, patients were classified as malnourished if the weight-for-age < 5th percentile, height-for-age < 5th percentile, or BMI-for-age < 10th percentile [8,20]. Although mean anthropometric values were within reference ranges, 38% of children were underweight by BMI-for-age, and 48% were underweight by weight percentile. No participants were classified as overweight or obese. These findings indicate a substantial burden of undernutrition in the studied cohort, although direct comparison with population-level Palestinian CF data is limited by the absence of comparable registry data. Underweight is very common for CF patients; it usually occurs due to chronic infections, malabsorption, and an imbalance between energy intake and energy demand. Frequent use of antibiotics is often associated with limiting nutrient intake and causing anorexia in CF patients [15]. Moreover, inadequate airway clearance can cause mucus production that can be swallowed and contributes in increasing satiety and reducing appetite. Intestinal obstructive syndrome and constipation can cause symptoms like abdominal pain and bowel obstruction that can lead to reduced intake [21]. In some cases, undernutrition has been linked with patients’ long stays in the hospital [22]. Whatever the reason is behind the inadequate dietary intake, dietary assessment and therapeutic intervention are essential for underweight patients to increase their body weight. The negative impact of underweight in the long term is proven, especially on lung function. Previous studies have shown that lung function and nutrition are co-dependent variables in CF [23].

Assessment of pulmonary function is important; it allows identification of patients with pulmonary dysfunction. Forced expiratory volume in 1 s is considered a standard spirometric measure for the management and assessment of cystic fibrosis lung disease; normal FEV1 is ≥80% [24]. In this study, almost half of the patients had abnormal levels of FEV1, and that’s not a good sign since there is a strong association between pulmonary function and nutritional status [25]. The results showed that about 77% of the patients had abnormal levels of 25(OH)D. Therefore, a good nutritional status should be improved and maintained to have better laboratory results and respiratory status [26]. Pancreatic insufficiency and fat malabsorption put CF patients at risk for fat-soluble vitamin deficiency, including 25(OH)D; it has been shown by various studies that vitamin D deficiency is common even with supplementation [27]. The prevalence of inadequate vitamin D levels in the North American pediatric CF population has been reported to be 95% [28]. The lower prevalence observed here than in some North American reports may reflect differences in sample characteristics, clinical management, supplementation, pancreatic function, measurement availability, or study setting.

Fat malabsorption can persist in CF even with pancreatic enzyme replacement therapy; therefore, dietary fat intake and enzyme adequacy should be considered together when evaluating energy and nutrient absorption [29]. High-energy, high-fat diets accompanied by pancreatic enzyme replacement therapy have been considered a standard for CF patients’ nutritional care to improve health and quality of life [1]. The nutritional guidelines according to the European Food Safety Authority (EFSA) recommended that CF children consume 35–40% of their caloric intake from fat to improve survival and achieve energy balance [14]. The guidelines focused on meeting body weight for age and stature for age by increasing the intake of fat. Essential fatty acid abnormalities are common in children and infants with CF, and loss of lean body mass is reported in adolescents with CF. Therefore, it is important to increase dietary intake of fatty acids and protein [16].

In our study, based on the three-days record, fat intake was very close to the recommended level, but it should be noted that the intake of saturated fatty acids was higher than unsaturated fatty acids, which puts patients at risk of developing cardiovascular diseases. Attention must be taken when choosing the type of fat that improves the patient’s health without putting the patient at risk of having other diseases. However, there was an inadequate intake of protein compared to the recommendation. In regard to carbohydrate intake, patients had a higher intake level than the recommendation. The higher proportion of energy derived from carbohydrate may reflect the dietary patterns of the participating children; however, the present study did not formally quantify food categories or sources of carbohydrate, so this explanation remains speculative. Our result regarding macronutrients intake was in agreement with Collins (2018), who found that patients who consume poor quality diet and meets their energy requirements through large amount of carbohydrates, might not achieve adequate protein intakes [30].

Patients with CF have higher requirements for calcium, sodium, zinc, iron, and fat-soluble vitamins because of intestinal malabsorption, chronic inflammation, and increased sweating that are common [16]. In our study, based on the three-day record, the youngest age group had the highest adherence to the recommendations, probably due to increased parental supervision and increased nutrient requirements as they get older. Shakkottai et al. (2015) conducted a study on 204 CF patients aged 0–21 years to check their adherence level and found similar results [31]. Calcium is one of the essential nutrients and must be taken from food. The main function of calcium is to build strong teeth, bones, and muscles [32]. Calcium in CF patients can be lower than in normal people due to the deficiency of 25(OH) D and gastrointestinal malabsorption of calcium [33]. Calcium intake was significantly below the selected reference values in the 9–13- and 14–18-year groups, whereas sodium and iron intake did not differ significantly from their respective reference values. These dietary findings should not be interpreted as evidence of biochemical deficiency because biochemical status for these nutrients was not assessed. The reason behind this decrease in calcium intake might be the dietary pattern changes. Children start to choose the food they like and avoid the kinds of food that are high in calcium by substituting dairy beverages with soda, for example [34]. Calcium-rich foods and drinks like milk, cheese, ice cream, and yogurt are recommended to achieve their dietary goals. Calcium intake should be assessed at least yearly [16]. Genotype, pancreatic exocrine function/pancreatic insufficiency status, and detailed pancreatic enzyme replacement therapy (PERT) dosing or adequacy data were not available in the present dataset. Consequently, the observed nutrient intake patterns cannot be attributed specifically to malabsorption, pancreatic insufficiency, genotype, disease severity, or PERT adequacy.

Inadequate levels of sodium are common in patients with CF due to the excessive salt loss through the skin (sweating). There is a high risk for sodium loss in the summer due to rapid breathing, fever, and fluid loss because of vomiting or diarrhea [35]. Sodium deficiency can lead to impaired growth in infants [36]. In this study, patients’ intake of sodium for all age groups was similar to recommendations for healthy children according to the Food and Nutrition Board, Institute of Medicine, National Academies; because CF-specific sodium targets were not consistently available in the source dataset, sodium intake was compared with the selected pediatric reference value and should not be interpreted as a definitive assessment of CF-specific sodium adequacy. This is probably due to adolescent patients tending to eat salty food such as crackers, chips, and fast food, which may increase their sodium intake dramatically. Similarly, iron and zinc were compared with general pediatric reference values because CF-specific targets were not available in the source dataset.

In our study, iron and zinc intake for the three age groups was adequate compared to the recommendations for healthy children according to the Food and Nutrition Board, Institute of Medicine, National Academies (i.e., there were no specific iron and zinc recommendations for CF children). It is preferred if patients have at least yearly blood tests to check their iron and zinc level, and increase their daily intake to avoid any future deficiencies. Even well-nourished patients are at risk of iron deficiency [37].

One objective was to assess caregiver CF-specific nutritional knowledge and separately evaluate patient adherence to recommended nutritional practices. In our study, thirty percent of caregivers had poor CF-specific nutritional knowledge, whereas only 12% achieved a very-good score. This distribution suggests an opportunity for structured, culturally appropriate nutritional education. However, because caregiver knowledge was not significantly associated with the measured clinical and biochemical outcomes in this study, these findings should not be interpreted as evidence of a direct causal effect. It was found that most of the participating parents had primary and secondary education levels, and our results revealed that the higher the school education level, the better the nutritional knowledge for parents. Sheehan et al. [38] confirmed this finding and revealed that a high level of parental nutritional knowledge and awareness can decrease many complications. Intensive training for parents about CF management techniques can help them improve their nutritional knowledge level; those trainings showed improvements in patients’ nutritional status in agreement with previous studies that were conducted by Stark et al. [39]. Routine clinic visits provide the parents with the opportunity to speak with the health care professionals about nutrition and increase their nutritional knowledge [40].

The second aim of the study was to assess patients’ adherence to the recommendations of the dietitian and doctor regarding a high-fat, high-calorie diet and doses of pancreatic enzyme replacement therapy. Although a high percentage of our patients had good adherence to nutritional recommendations (90%). The mean percentage of patients’ adherence to nutritional recommendations was (58.57%); poor adherence to the nutritional recommendations was reported in previous studies of children with CF ranging between 16–20% [41]. In most cases, patient’s adherence percentage is associated with parental knowledge, involvement in the therapy, and communication with their CF children, which helps them understand the importance of their treatment and diet [19]. Exploring ways of increasing attention to diet from both children and parents at home could be a strategy to assist in preventing growth failure in these people [40]. Higher patient adherence was associated with higher FEV1% predicted and serum albumin. However, the cross-sectional design cannot determine whether greater adherence contributes to better clinical status or whether children with better clinical status are more able to adhere to recommendations. In addition, the analysis was based on a small sample from a single specialized hospital, limiting statistical precision and generalizability. The findings are most directly relevant to the studied Palestinian/West Bank clinical setting and should not be assumed to represent all children with CF. The most common strategies used by health professionals to improve patients’ dietary adherence were educational counseling with behavioral and psychological interventions that can improve adherence [19].

Adherence to nutritional recommendations is an important component of pediatric CF care. Families and caregivers contribute substantially to treatment implementation and self-management [42]. However, the present cross-sectional study cannot determine whether better adherence improves clinical status or whether children with better clinical status are more able to adhere to recommendations.

Clinically, the findings support continued nutritional screening of children with CF, individualized dietary counseling, monitoring of micronutrient status, and structured education for caregivers and children. Particular attention may be warranted for children with low BMI-for-age, suboptimal protein or calcium intake, or inadequate 25(OH)D concentrations. These implications should be interpreted as supportive of targeted care rather than evidence that the interventions would necessarily improve outcomes, given the observational design.

### Limitations

This study has several limitations. The cross-sectional design precludes causal inference, and the small single-center sample limits generalizability. Dietary intake was assessed over only three days and may be affected by day-to-day variation, portion-size estimation, and reporting error. The caregiver nutritional knowledge questionnaire was developed specifically for this study and underwent expert content review; its broader psychometric properties require further evaluation. Clinical and biochemical measurements were unavailable for some participants, resulting in variable denominators. In addition, some dietary reference values were based on general pediatric recommendations where CF-specific values were unavailable. Multiple comparisons were also performed, so findings with *p*-values close to 0.05 should be interpreted cautiously. In addition, genotype, pancreatic exocrine function/pancreatic insufficiency status, and detailed PERT dosing or adequacy were not available, limiting interpretation of dietary differences in relation to malabsorption and disease severity. Also, further studies should evaluate the reliability and construct validity of the instruments used in this study. The incomplete availability of FEV1 and biochemical measurements also introduces uncertainty regarding the representativeness of these outcomes. Finally, multiple comparisons were conducted without adjustment for multiplicity, and several subgroup analyses involved small sample sizes; therefore, findings with *p*-values close to 0.05 should be interpreted cautiously.

## 5. Conclusions

In this single-center cohort of children with CF, undernutrition was common, with 38% classified as underweight by BMI-for-age and 48% by weight-for-age percentile. Protein intake was significantly below the selected reference value, and calcium intake was significantly below the selected reference values in children aged 9–13 and 14–18 years. Among children with available measurements, 48% had FEV1 < 80% predicted and 76.9% had 25(OH)D concentrations < 30 ng/mL. Higher patient dietary adherence was associated with higher FEV1% predicted and serum albumin concentrations, whereas caregiver nutritional knowledge was not significantly associated with the measured clinical or biochemical outcomes. These findings support regular nutritional assessment, individualized dietary counseling, micronutrient monitoring, and structured education for children and caregivers, while larger longitudinal and multicenter studies are needed to confirm these associations. These findings should be interpreted as observational associations rather than evidence that caregiver knowledge or adherence causes differences in clinical outcomes.

## Figures and Tables

**Table 1:** Patients’ general characteristics (*N* = 50).

Patients Characteristics	*n* (%)
Age	
4–8 years	20 (40)
9–13 years	20 (40)
14–18 years	10 (20)
Sex	
Girls	29 (58)
Boys	21 (42)
BMI percentile	
Normal	31 (62)
Underweight	19 (38)
Weight percentile	
Normal	26 (52)
Underweight	24 (48)
Stature percentile	
Normal	37 (74)
Under-height	13 (26)

Values are presented as frequency and %. Note: BMI was interpreted using age- and sex-specific NCHS references. No participants were classified as overweight or obese. Genotype, pancreatic exocrine status, PERT adequacy, and CFTR-modulator use were not available in the study dataset.

**Table 2:** Patients’ clinical and biochemical tests (*N* = 50).

Clinical & Biochemical Tests	*n* (%)
FEV1	
Normal ≥ 80%	13 (52)
Abnormal	12 (48)
Total	25 (100)
25(OH)D	
Normal (≥30 ng/mL)	9 (23.1)
Insufficiency (20–29 ng/mL)	10 (25.6)
Deficiency (≤20 ng/mL)	20 (51.3)
Total	39 (100)
Total protein	
Normal (6–8.3g/dL)	35 (92.1)
Abnormal	3 (7.9)
Total	38 (100)
Albumin	
Normal (3.5–5.0 g/dL)	39 (100)
Abnormal	0 (0)
Total	39 (100)

Values are presented as frequency and %. 25 (OH) D deficiency: (≤20 ng/mL), 25 (OH) D insufficiency: (21–29 ng/mL). 25 (OH) D normal: (≥30 ng/mL). Total protein deficiency: (<6 g/dL); total protein normal: (6–8.3 g/dL). Albumin deficiency: (<3.5 g/dL); Albumin normal: (3.5–5.0 g/dL). Note: Percentages are calculated using participants with available measurements. Albumin and total protein are presented as routinely available biochemical measures and are not interpreted as specific nutritional-status markers.

**Table 3:** Parents’ demographic characteristics (*N* = 50).

Parent’s Characteristics	*n* (%)
Sex	
Females	50 (100)
Males	0 (0)
Educational level	
Illiterate	1 (2)
Primary	25 (50)
Secondary	17 (34)
College	7 (14)
Relatives vs. non-relatives parents	
First-degree relatives	29 (58)
Second-degree relatives	8 (16)
Non-relatives	13 (26)
Family history of CF	
Yes	19 (38)
No	31 (62)
Number of kids with CF	
One kid	28 (56)
More than one child with CF	22 (44)

Values are presented as frequency and %. Note: All participating caregivers were mothers. Maternal CF diagnosis was not recorded in the study dataset.

**Table 4:** Mean percentage of daily dietary intake of macronutrients and the recommended levels for CF patients.

Macronutrients	Intake (%) Mean ± SD	Recommended Level for CF Patients	*p*-Value
Fat (%)	38.42 ± 7.04	40	0.12
Protein (%)	17.72 ± 4.68	20	0.01
Carbohydrates (%)	51.17 ± 8.93	40	0.01

Values are presented as mean ± SD. *p*-value is considered significant if *p* ≤ 0.05.

**Table 5:** Mean Daily Intakes of Calcium, Sodium, and Zinc Compared to Recommended Levels for CF Patients.

Boys + Girls	Calcium Intake (mg) Mean ± SD	Recommended Level (mg)	*p*-Value
4–8 years	750.62 ± 297.95	800	0.46
9–13 years	598.91 ± 285.23	1000	0.01
14–18 years	831.49 ± 216.56	1150	0.01
Boys + Girls	Sodium Intake (mg) Mean ± SD	Recommended Level (mg)	*p*-Value
4–8 years	1943.98 ± 818.97	1800	0.44
9–13 years	2165.01 ± 936.33	2400	0.27
14–18 years	2899.04 ± 1138.50	2400	0.19
Boys	Zinc Intake (mg) Mean ± SD	Recommended Level (mg)	*p*-Value
4–8 years	6.19 ± 3.28	5	0.17
9–13 years	7.83 ± 2.44	8	0.05
14–18 years	7.20 ± 1.51	11	0.05
Girls	Zinc Intake (mg) Mean ± SD	Recommended Level (mg)	*p*-Value
4–8 years	6.19 ± 3.28	5	0.18
9–13 years	5.25 ± 2.66	8	0.05
14–18 years	7.08 ± 4.23	9	0.03

Values are presented as mean ± SD. *p*-value is considered significant if *p* ≤ 0.05. Note: CF-specific reference values were used where available in the source dataset; general pediatric reference values were retained where CF-specific recommendations were unavailable.

**Table 6:** Mean dietary intake of iron and the recommended level for healthy children.

Boys	Iron Intake (mg) Mean ± SD	Recommended Level/DRI	*p*-Value
4–8 years	9.05 ± 3.67	10	0.43
9–13 years	11.36 ± 5.03	8	0.16
14–18 years	15.05 ± 2.36	11	0.16
Girls			
4–8 years	10.85 ± 4.13	10	0.52
9–13 years	9.04 ± 2.77	8	0.18
14–18 years	12.28 ± 7.18	15	0.44

Values are presented as mean ± SD. *p*-value is considered significant when it is ≤0.05. Note: General pediatric DRI values were used because CF-specific iron recommendations were not available in the source dataset.

**Table 7:** Level of parents’ nutritional knowledge and patients’ adherence to nutritional recommendations.

**Level of Parents’ Nutritional Knowledge**	***n* (%)**
Poor (<40%)	15 (30)
Good (40–60%)	29 (58)
Very good (>60%)	6 (12)
* Mean % ± SD	44.33 ± 15.37
**Level of Patient Adherence to Nutritional Recommendations**	***n* (%)**
Poor (<40%)	5 (10)
Good (40–60%)	27 (54)
Very good (>60%)	18 (36)
* Mean % ± SD	58.57 ± 16.64

Values are presented as frequency and %. * Values are presented as mean ± SD. Note: Knowledge and adherence categories are study-specific descriptive classifications and are not validated clinical thresholds.

**Table 8:** Association between parents’ nutritional knowledge levels, patients’ adherence to nutritional recommendations, and clinical/biochemical tests.

**Parental Nutritional Knowledge**	**FEV1** **Mean ± SE**	**25(OH)D** **Mean ± SE**	**Total Protein** **Mean ± SE**	**Albumin** **Mean ± SE**
Poor	76.11 ± 11.33 ^a^	19.79 ± 3.99 ^a^	7.56 ± 0.22 ^a^	4.02 ± 0.16 ^a^
Good	83.76 ± 4.82 ^a^	23.02 ± 2.23 ^a^	7.15 ± 0.15 ^a^	4.14 ± 0.05 ^a^
Very good	82.33 ± 2.4 ^a^	18.82 ± 2.80 ^a^	7.63 ± 0.15 ^a^	4.42 ± 0.08 ^a^
*p*-value	0.76	0.61	0.18	0.11
**Patient’s Adherence to Nutritional Recommendations**	**FEV1** **Mean ± SE**	**25(OH)D** **Mean ± SE**	**Total Protein** **Mean ± SE**	**Albumin** **Mean ± SE**
Poor	51.00 ± 19.00 ^a^	17.00 ± 5.10 ^a^	6.94 ± 0.57 ^a^	3.55 ± 0.50 ^a^
Good	84.07 ± 5.72 ^b^	24.38 ± 2.67 ^a^	7.39 ± 0.13 ^a^	4.19 ± 0.06 ^b^
Very good	86.37± 6.03 ^b^	18.67 ± 2.18 ^a^	7.28 ± 0.22 ^a^	4.21 ± 0.07 ^b^
*p*-value	0.05	0.22	0.66	0.02

Values are presented as frequency and %. *p*-value is considered significant if *p* ≤ 0.05. Values with different superscripts letter within the same row differ significantly.

## Data Availability

The original contributions presented in the study are included in the article; further inquiries can be directed to the corresponding author.

## References

[1] Schindler T., Michel S., Wilson A.W. (2015). Nutrition management of cystic fibrosis in the 21st century. Nutr. Clin. Pract..

[2] Van Devanter D.R., Kahle J.S., O’Sullivan A.K., Sikirica S., Hodgkins P.S. (2016). Cystic fibrosis in young children: A review of disease manifestation, progression, and response to early treatment. J. Cyst. Fibros..

[3] Kelly J. (2017). Environmental scan of cystic fibrosis research worldwide. J. Cyst. Fibros..

[4] Salah S., Rumman N., Nassar A., Khdour M., Hallak H. (2023). Clinical characteristics and outcomes of cystic fibrosis in Palestine: Cross sectional study. Pediatr. Pulmonol..

[5] Savant A., Lyman B., Bojanowski C., Upadia J. (2024). Cystic Fibrosis. GeneReviews^®^.

[6] Farrell P.M., White T.B., Howenstine M.S., Munck A., Parad R.B., Rosenfeld M., Sommerburg O., Accurso F.J., Davies J.C., Rock M.J. (2017). Diagnosis of cystic fibrosis in screened populations. J. Pediatr..

[7] Leeman J., Sandelowski M., Havill N.L., Knafl K. (2015). Parent-to-child transition in managing cystic fibrosis: A research synthesis. J. Fam. Theory Rev..

[8] Engelen M.P., Schroder R., Van der Hoorn K., Deutz N.E., Com G. (2012). Use of body mass index percentile to identify fat-free mass depletion in children with cystic fibrosis. Clin. Nutr..

[9] Cystic Fibrosis Foundation (2026). Understanding Changes in Life Expectancy.

[10] Farrell P.M., White T.B., Ren C.L., Hempstead S.E., Accurso F., Derichs N., Howenstine M., McColley S.A., Rock M., Rosenfeld M. (2017). Diagnosis of cystic fibrosis: Consensus guidelines from the Cystic Fibrosis Foundation. J. Pediatr..

[11] McDonald C.M., Alvarez J.A., Bailey J., Bowser E.K., Farnham K., Mangus M., Padula L., Porco K., Rozga M. (2020). Academy of Nutrition and Dietetics Evidence Analysis Center Academy of Nutrition and Dietetics: 2020 Cystic Fibrosis Evidence Analysis Center Evidence-Based Nutrition Practice Guideline. J. Acad. Nutr. Diet..

[12] Struyvenberg M.R., Martin C.R., Freedman S.D. (2017). Practical guide to exocrine pancreatic insufficiency–breaking the myths. BMC Med..

[13] Culhane S., George C., Pearo B., Spoede E. (2013). Malnutrition in cystic fibrosis: A review. Nutr. Clin. Pract..

[14] Engelen M.P., Com G., Deutz N.E. (2014). Protein is an important but undervalued macronutrient in the nutritional care of patients with cystic fibrosis. Curr. Opin. Clin. Nutr. Metab. Care.

[15] Wilschanski M., Munck A., Carrion E., Cipolli M., Collins S., Colombo C., Declercq D., Hatziagorou E., Hulst J., Kalnins D. (2024). ESPEN-ESPGHAN-ECFS guideline on nutrition care for cystic fibrosis. Clin. Nutr..

[16] Turck D., Braegger C.P., Colombo C., Declercq D., Morton A., Pancheva R., Robberecht E., Stern M., Strandvik B., Wolfe S. (2016). ESPEN-ESPGHAN-ECFS guidelines on nutrition care for infants, children, and adults with cystic fibrosis. Clin. Nutr..

[17] Powers S.W., Mitchell M.J., Patton S.R., Byars K.C., Jelalian E., Mulvihill M.M., Hovell M.F., Stark L.J. (2005). Mealtime behaviors in families of infants and toddlers with cystic fibrosis. J. Cyst. Fibros..

[18] Coyne I., Malone H., Chubb E., While A.E. (2018). Transition from pediatric to adult healthcare for young people with cystic fibrosis: Parents’ information needs. J. Child Health Care.

[19] Bishay L.C., Sawicki G.S. (2016). Strategies to optimize treatment adherence in adolescent patients with cystic fibrosis. Adolesc. Health Med. Ther..

[20] Lai H.C., Kosorok M.R., Sondel S.A., Chen S.T., Fitz Simmons S.C., Green C.G., Shen G., Walker S., Farrell P.M. (1998). Growth status in children with cystic fibrosis based on the National Cystic Fibrosis Patient Registry data: Evaluation of various criteria used to identify malnutrition. J. Pediatr..

[21] Solomon M., Bozic M., Mascarenhas M.R. (2016). Nutritional issues in cystic fibrosis. Clin. Chest Med..

[22] Chima C.S., Barco K., Dewitt M.L., Maeda M., Teran J.C., Mullen K.D. (1997). Relationship of nutritional status to length of stay, hospital costs, and discharge status of patients hospitalized in the medicine service. J. Am. Diet. Assoc..

[23] Steinkamp G., Wiedemann B. (2002). Relationship between nutritional status and lung function in cystic fibrosis: Cross sectional and longitudinal analyses from the German CF quality assurance (CFQA) project. Thorax.

[24] König P., Ner Z., Acton J.D., Ge B., Hewett J. (2018). Is an FEV1 of 80% predicted a normal spirometry in cystic fibrosis children and adults. Clin. Respir. J..

[25] Kerem E., Viviani L., Zolin A., MacNeill S., Hatziagorou E., Ellemunter H., Drevinek P., Gulmans V., Krivec U., Olesen H. (2014). Factors associated with FEV1 decline in cystic fibrosis: Analysis of the ECFS patient registry. Eur. Respir. J..

[26] Benjamin D.R. (1989). Laboratory tests and nutritional assessment: Protein-energy status. Pediatr. Clin. N. Am..

[27] Green D., Carson K., Leonard A., Davis J.E., Rosenstein B., Zeitlin P., Mogayzel J.P. (2008). Current treatment recommendations for correcting vitamin D deficiency in pediatric patients with cystic fibrosis are inadequate. J. Pediatr..

[28] Norton L., Page S., Sheehan M., Mazurak V., Brunet-Wood K., Larsen B. (2015). Prevalence of inadequate vitamin d status and associated factors in children with cystic fibrosis. Nutr. Clin. Pract..

[29] Figueroa V., Milla C., Parks E.J., Schwarzenberg S.J., Moran A. (2002). Abnormal lipid concentrations in cystic fibrosis. Am. J. Clin. Nutr..

[30] Collins S. (2018). Nutritional management of cystic fibrosis–an update for the 21st century. Pediatr. Respir. Rev..

[31] Shakkottai A., Kidwell K.M., Townsend M., Nasr S.Z. (2015). A five-year retrospective analysis of adherence in cystic fibrosis. Pediatr. Pulmonol..

[32] Beto J.A. (2015). The role of calcium in human aging. Clin. Nutr. Res..

[33] Gronowitz E., Garemo M., Lindblad A., Mellström D., Strandvik B. (2003). Decreased bone mineral density in normal-growing patients with cystic fibrosis. Acta Paediatr..

[34] Miller G.D., Jarvis J.K., McBean L.D. (2001). The importance of meeting calcium needs with foods. J. Am. Coll. Nutr..

[35] Sermet-Gaudelus I., Mayell S.J., Southern K.W. (2010). Guidelines on the early management of infants diagnosed with cystic fibrosis following newborn screening. J. Cyst. Fibros..

[36] Coates A.J., Crofton P.M., Marshall T. (2009). Evaluation of salt supplementation in CF infants. J. Cyst. Fibros..

[37] Uijterschout L., Nuijsink M., Hendriks D., Vos R., Brus F. (2014). Iron deficiency occurs frequently in children with cystic fibrosis. Pediatr. Pulmonol..

[38] Sheehan J., Massie J., Hay M., Jaffe A., Glazner J., Armstrong D., Hiscock H. (2012). The natural history and predictors of persistent problem behaviours in cystic fibrosis: A multicentre, prospective study. Arch. Dis. Child..

[39] Stark L.J., Powers S.W., Jelalian E., Rape R.N., Miller D.L. (1994). Modifying problematic mealtime interactions of children with cystic fibrosis and their parents via behavioral parent training. J. Pediatr. Psychol..

[40] Anthony H., Paxton S., Bines J., Phelan P. (1999). Psychosocial predictors of adherence to nutritional recommendations and growth outcomes in children with cystic fibrosis. J. Psychosom. Res..

[41] Modi A.C., Lim C.S., Yu N., Geller D., Wagner M.H., Quittner A.L. (2006). A multi-method assessment of treatment adherence for children with cystic fibrosis. J. Cyst. Fibros..

[42] McDonald C.M., Haberman D., Brown N. (2013). Self-efficacy: Empowering parents of children with cystic fibrosis. J. Cyst. Fibros..

